# Annotations

**Published:** 1889-06-08

**Authors:** 


### Annotations.

**An Open-Air Academy.**

The morning train in the latter part of this month of May affords rare opportunities for those who live, for the summer, thirty or forty miles out of London. The dwellers at Tunbridge Wells, Brighton, and other towns on or near the South Coast, are specially privileged. It is true that the majority are no sooner seated than they at once commence the worrying and unhealthy study of the politics or finance of the day. But the majority have got no eyes, and the finest pictures appeal to them in vain. A walk through the Academy may give greater variety than a thirty-mile ride in the sunny South; but what can it offer in beauty compared with the spreading green of the Kentish oaks, the brilliant yellow of the gorse and broom, the snowy pink of the apple orchards, and the hundred mingling colours of the fields, the gardens, and the woods? In every mile of the journey a dozen different subjects offer themselves to the artist's brush and seem to say, "Can you put on your canvas anything so fresh and beautiful as I am?" In the higher parts of Kent innumerable little pools, each surrounded by its shrubs and wild flowers, or sometimes by forest trees, form a conspicuous feature of the landscape, and give it a character peculiarly its own. Here and there an ancient church with its square Saxon tower, red-tiled roof, and grey, ivy-covered walls, carries the mind back to days when London was but a tithe of its present size, and when the want of railways kept it and its inhabitants strictly within the limits which Nature had assigned to them. For six weeks past the rooks have been cawing in the budding trees, and all the song-birds have been busy with their nests. Even as the train rushes past, pairs of happy partridges may be seen in almost every grass field, and here and there the lovely plumage of the pheasant shows itself under the edges of the hanging wood. The young rabbits are beginning to put on their boldest airs, and to sit on their haunches with ears erect, watching the train rush by, as if they would say "Who cares for you?" The parks, the home-farms, the cottages, with their gardens of springing peas and bright sweet-scented flowers, the cattle grazing or lying down in the shade, the steady plough-horses and steadier men, the women in the hop fields tying up the "byne," the groups of children hastening to school, half-thinking of playing truant by the way—these and a hundred other pictures constitute an Open-Air Academy, with which Piccadilly competes in vain. Happy is the man who has clearness of eye and healthful simplicity of mind to enjoy these works of Nature—the oldest, as she is surely the greatest, of all the "Old Masters."

**A Modern Cain.**

Civilised society in this country, apparently, has much sympathy with Cain in its persistent determination not to recognise any responsibility for the murder of helpless children. Dr. Macdonald, M.P., last week, stated certain facts before the Select Committee on Friendly Societies, relative to infantile mortality and life assurance, which are more than startling, and demand instant action on the part of those who hold that a Christian nation is bound to have a conscience. Dr. Macdonald is a Member of Parliament and Coroner for North-East London, and in both these capacities it may be assumed that he spoke with a full sense of reponsibility. He had come to the conclusion, he said, from circumstances known personally to himself, that infantile assurance was a temptation and almost an incentive to neglect and even to murder. Probably every reader of newspapers will admit that children are sometimes done to death by parents and others in order to obtain possession of insurance money. But what the public do not seem to realise is the extent to which this unnatural and murderous practice prevails. Let every man and woman of conscience and feeling consider a few of Dr. Macdonald's figures, collected by his own officer, and vouched for by himself. According to these, of 262 young children on whom inquests had been held during six months, no fewer than 126 were insured in life offices. Five of these insured little ones were under a month old, so early do some parents begin to speculate on their baby's premature death. Twenty-two were above one and under three months old; twenty-four over three and under six months; twenty-six over six and under twelve months, and so on, the ratio of insured children to uninsured steadily increasing with age. It is surely a very startling fact that of all the children on whom coroner's inquests had been held during a definite period, nearly half should have brought money to their parents or guardians by their untimely death. It is still more startling to find that when we get past the age of three months a very much larger proportion than half of the children who have died under doubtful circumstances have been insured; at one age, indeed, the number of insured was nearly twice that of the uninsured. Can these numerous deaths of insured children be accounted for by natural causes? It is probable that the proportion of living insured children to living non-insured is not more than one in twenty in Dr. Macdonald's district, even if it be so much; whilst the proportion of insured to non-insured among those who die doubtful deaths is almost one in two, and at certain ages almost two in three. Even outsiders may see the extreme probability—amounting to certainty—that this enormous proportion of insured children cannot come by their deaths fairly. But Dr. Macdonald, looking at the facts from within, and speaking with the sobriety that becomes his responsible position, is convinced that "infantile assurance is a temptation and almost an incentive to neglect, and even to murder." In other words, a distinct premium is put upon the cruel butchery of helpless babies and young children, and nobody seems to mind. The first murderer who had killed one grown-up man refused to admit that he was his brother's keeper. If Dr. Macdonald's inferences as to his limited district be correct, young children must be murdered by the score in all the districts of this vast London every year; and yet we all fold our hands and do nothing. Are we so very much better than Cain?

**The Dangerous Season.**

Summer is *par excellence* the dangerous season, so far as food is concerned. A note of warning was given last week by the sudden and alarming death of Edward Dell, a milk-carrier, of Wellington-road, Kentish Town. Dell's wife bought a mackerel on Friday, which she cooked on Saturday morning, she and her husband breakfasting off it. Mrs. Dell did not suffer; but Dell appears to have eaten the part of the fish nearest the head, including, probably, the gills. The day after he was seized with inflammation of the stomach, and, becoming delirious, escaped from the house, and wandered about Hampstead for a considerable time. When found he was taken to the St. Pancras Infirmary, where he died on the following Tuesday. It is known to everybody that food decomposes very rapidly in hot weather. Of all meat foods fish is the soonest to become bad, and of all kinds of fish mackerel appears to be decidedly the worst. It is a good rule not to eat mackerel at all in the summer, except at the seaside. Even there it should only be purchased by those who are judges of its freshness, and it should always be cooked on the very day it is bought. A great many persons are made ill by mackerel who are not poisoned outright. It is desirable also to repeat the warning given every summer against unripe and too ripe fruit, bad ices, stale eggs, and milk that has begun to "turn." Of course, the cases that end fatally as the result of partaking of unwholesome food are not, perhaps, very numerous, but those who are made seriously ill with various pains, sickness, and diarrhœa may be numbered by the hundred. What is required is strict care and supervision on the part of the mother or other responsible person. For though few die of bad food, yet we never know what husband, or wife, or child will be the particular one who shall eat the dangerous portion of the fish, or the most rotten of the gooseberries, or who, being very susceptible or in bad health at the moment, shall be the unfortunate victim. Mistresses should teach their servants and parents their children that all unsound food is bad and dangerous, and should never, for any reason, be eaten. Even the very poor had better endure the pangs of hunger than run the risk of pain and death by eating unwholesome food.

